# Anxiety disorders: a cross-sectional study of clinical and biological associations

**DOI:** 10.1007/s00702-026-03169-1

**Published:** 2026-06-13

**Authors:** Jessica L. Fernandes, Graziela Amboni, Camila O. Arent, Lucas C. Pedro, Gabriel S. Mondo, Flavia S. Niero, Josimar G. Pereira, Margarete D. Bagatini, Zuleide Maria Ignácio, Luciane B. Ceretta, Gislaine Z. Réus

**Affiliations:** 1 Translational Psychiatry Laboratory, Graduate Program in Health Sciences, University of Southern Santa Catarina (UNESC), Criciúma, SC 88806-000 Brazil; 2 Laboratory of Physiology, Pharmacology, and Psychopathology, Graduate Program in Biomedical Sciences, Federal University of the Southern Frontier, Chapecó, SC Brazil; 3 Graduate Program in Public Health, University of Southern Santa Catarina (UNESC), Criciúma, SC Brazil

**Keywords:** Psychiatric disorders, Biological rhythms, Oxidative stress, Antioxidants, Anxiety disorders

## Abstract

It is estimated that more than 300 million people worldwide suffer from anxiety disorders. Oxidative stress is increasingly recognized as a factor in the development and progression of anxiety disorders. This study aimed to investigate the association of psychological symptoms and oxidative stress parameters with anxiety disorders. This is a cross-sectional study that included a paired sample of adults diagnosed with anxiety disorders (116 individuals) and without a diagnosis of anxiety disorders (234 individuals). Data collection took place from September 2020 to July 2021 during the COVID-19 pandemic. We assessed the presence of psychiatric conditions using standardized diagnostic criteria, the Hamilton Rating Scale for Anxiety (HAM-A), the Hamilton Rating Scale for Depression (HAM-D), the Biological Rhythms in Neuropsychiatry Assessment Interview (BRIAN), and the Functional Assessment Short Test (FAST). Stress levels were assessed using the Stress Symptom Inventory. Oxidative stress parameters were measured in the serum sample. Individuals with anxiety disorders had higher levels of stress, greater severity of depressive and anxiety symptoms, impaired cognitive functioning, and greater impairment in biological rhythms compared with individuals without anxiety disorders. In addition, individuals with anxiety disorders had decreased total antioxidant capacity compared with individuals without anxiety disorders. However, after correcting for multiple testing, the oxidative status did not differ between groups. This study demonstrates that anxiety disorders are associated not only with increased psychological distress and functional impairment but also with biological alterations. These findings support an exploratory role of oxidative stress in the pathophysiology of anxiety disorders.

## Introduction

Excessive anxiety has become an increasingly common reality (Wu et al. 2024; Zhang et al. 2025). Globally, anxiety disorders are currently among the leading causes of illness and are considered by the World Health Organization to be the sixth major cause of global disability (Zhao et al. 2023). The main subtypes within this group of disorders include generalized anxiety disorder, panic disorder, social anxiety disorder, specific phobia, and agoraphobia (Nowak et al. 2023; Tadros et al. 2025; Huang et al. 2025). Post-traumatic stress disorder and obsessive–compulsive disorder are now categorized separately (O'Leary and Khan 2024; Tadros et al. 2025). Diagnostic criteria comprise excessive fear and anxiety, restlessness, difficulty concentrating, fatigue, irritability, muscle tension, and changes in sleep patterns (Chand, Marwaha 2023; Munir, Takov 2022. The intensity, duration, and functional impairment caused by symptoms are also considered in the diagnostic process (American Psychiatric Association 2022). Common physiological reactions include dizziness, breathlessness, thoracic discomfort, and tachycardia (Szuhany, Simon 2022).

From a physiological perspective, fear and anxiety share similar mechanisms (Grogans et al. 2023). While fear is an adaptive response to physical threats, anxiety involves subjective apprehension toward perceived challenges, leading to maladaptive levels of distress relative to contextual factors (Beckers et al. 2023; Ohi et al. 2025). Both activate the sympathetic nervous system, triggering the release of adrenaline and noradrenaline, which are followed by responses such as increased heart rate, sweating, and altered memory and focus, thereby enhancing the individual’s ability to react and protect themselves (Hamm 2019). In the fight-or-flight circuit, the amygdala plays a central role by detecting threats, processing emotions, and modulating the physical and psychological reactions associated with fear (Öhman 2005; Shackman et al. 2024). The hippocampus provides information about the threatening context and modulates cortisol release by the hypothalamus (Simic et al., 2021). The prefrontal cortex, in turn, inhibits both amygdala hyperactivation and hypothalamic activity (Meier, Schwabe 2024). Multiple neurotransmitters are also involved in this process. Especially, GABA and serotonin. Individuals with anxiety disorders have been shown to have significantly lower plasma GABA levels (Shutta et al. 2021), indicating reduced characteristic neuroinhibitory activity (Liu et al. 2022). Serotonin, in turn, plays a modulatory role in anxiety, emotional memory, and neurophysiological excitability (Romain Ligneul, Mainen 2023).

In fact, an impairment of prefrontal control over the amygdala is closely related to both anxiety and depression (Lei et al. 2025; Liu et al. 2020). Stressful events are known contributors to mental disorders (Knezevic et al. 2023), with repeated exposure increasing the risk of initial and recurrent depressive episodes by lowering stress sensitivity (Monroe, Harkness 2022). A preclinical study further showed that chronic stress activates a molecular cascade linked to anxiety, whereas inhibition of this pathway mitigated the disorder’s features (Pandey et al. 2024). It is worth noting that comorbidity between depression and anxiety is widely recognized (Liu et al. 2021). The DSM allows for the combined assessment of this symptomatology, either using specifiers or by treating both conditions (American Psychiatric Association 2022).

It should be emphasized that anxiogenic conditions increase the risk of developing diseases such as diabetes, for example (Jiang et al. 2020). This may be partly explained by the fact that individuals with anxiety tend to have unhealthy dietary habits (Basiri et al. 2023). High consumption of sugar, carbohydrates, and processed foods, common in this group, may contribute to this association (Peixoto et al. 2025; Wang et al. 2024). Psychological stressors may also promote insulin resistance, a central factor in the development of type 2 diabetes (Sharma et al. 2022). Another significant comorbidity is sleeping disturbance (Wang et al. 2026). Studies show that approximately 50% of individuals with anxiety experience sleep-related problems. Furthermore, research indicates that sleep deprivation exacerbates anxiety symptoms (Chellappan and Aeschbach, 2022).

Spalding and colleagues (2020) also demonstrated that cognitive performance is commonly affected by anxiety, since individuals with prominent anxiety traits tend to perform worse on memory-related tasks. Similarly, in individuals with heightened anxiety, seemingly threatening stimuli trigger a heightened state of hypervigilance, which may impair sustained concentration (Bar-Haim et al. 2007). Indeed, research supports the notion that individuals with anxiety disorders have greater difficulty ignoring harmful or potentially threatening stimuli (MacLeod et al. 2019). Anxiety, especially when combined with negative emotional content, can disrupt the efficiency of working memory, thereby impairing task performance and overall functioning. (Yang et al. 2021).

Research has also pointed out the relationship between oxidative stress and anxiety disorders. In preclinical studies, induced chronic stress affected the medial prefrontal cortex, leading to oxidative stress and consequent anxiety-like symptoms (Dang et al. 2022). Similarly, elevated cortisol levels led to excessive oxidative stress and anxiety-related behaviors (Camargo et al. 2018). In humans, studies have shown a possible causal relationship between oxidative stress and anxiety (Ciu et al. 2025). The likelihood of oxidative stress-induced DNA damage in patients with generalized anxiety disorder has also been reported, although further studies are needed to confirm these hypotheses (Oktay et al. 2024). This context highlights the potential involvement of oxidative stress, alongside depressive, anxious, and stress-related symptoms, in anxiety disorders. However, studies simultaneously examining psychological symptoms, functional impairment, biological rhythms, and oxidative stress parameters in individuals with anxiety disorders, particularly in comparison with matched individuals without anxiety disorders, remain limited. Therefore, this study aimed to investigate the association between psychological symptoms, functional and biological measures, and oxidative stress parameters in individuals with anxiety disorders compared to those without such disorders, in a paired sample assessed during the COVID-19 pandemic.

## Methods

### Study design

This study is a cross-sectional study, which includes individuals with anxiety disorders and individuals without anxiety disorders. This study was structured by the Strengthening the Reporting of Observational Studies in Epidemiology (STROBE) guidelines to ensure transparency and methodological rigor.

### Setting

The study took place between September 2020 and July 2021 in two locations: (1) Universidade do Extremo Sul Catarinense (UNESC, Criciúma, Brazil) and (2) Universidade Federal da Fronteira Sul (UFFS, Chapecó, Brazil). The Research Ethics and Human Research Committee approved the study at both institutions, under protocols 4,172,382 and 4,298,662, respectively. The study was only initiated after the participants signed the Free and Informed Consent Form.

### Participants

The individuals included in this study were selected from a larger, multicenter cohort study with the primary objective of assessing the impact of COVID-19 on mental health. Further details have been published previously (Azevedo Cardoso et al. 2023; Réus et al. 2023; Barichello et al. 2025; Pedro et al. 2025). From this large study, 350 individuals were evaluated and validated using instruments for diagnosing anxiety disorders.

### Demographic characteristics and psychiatric assessment

Participants completed a sociodemographic questionnaire that included questions about their gender, race, marital status, age, occupation, and years of education.

The diagnosis of anxiety disorders (panic disorder, agoraphobia, social anxiety, specific phobia, generalized anxiety disorder, obsessive–compulsive disorder, and posttraumatic stress disorder) was based on the Mini International Neuropsychiatric Interview Plus (M.I.N.I. Plus) (Amorim 2000)—the M.I.N.I. Plus is a standardized, rapid application diagnostic interview that explores the primary psychiatric disorders of Axis I. This scale is intended for the in-depth assessment of mental disorders throughout the lifespan in clinical and psychiatric research settings. The MINI Plus systematically explores all inclusion and exclusion criteria, as well as the chronology (onset and duration of disorders and the number of episodes) for 23 diagnostic categories in the DSM-5.

The severity of depressive symptoms was assessed using the Hamilton Depression Rating Scale (HAM-D). The instrument consists of 17 questions, which are quantitatively classified according to symptom severity. The total score yields a discrete variable, with higher scores indicating greater severity (Hamilton 1967).

The severity of anxiety symptoms was assessed using the Hamilton Anxiety Rating Scale (HAM-A), which consists of 14 items. Each item is scored according to its intensity, and the total score is the sum of the points assigned to all items, ranging from 0 to 56. Higher total scores indicate greater symptom severity (Hamilton 1959).

Stress levels were assessed using the Stress Symptom Inventory. The inventory consists of an adaptation of items from the Checklist-90-R Symptom Inventory (SCL-90-R) (Derogatis 1994). The SCL-90-R consists of 90 items, of which 24 were selected, with item scores ranging from 0 to 5 (Bertollo et al. 2020). Items expressing stress symptoms were selected and compared with stress items from the SCL-90-R (Lipp et al. 1994). The items identified were related to anxiety, depression, hostility, and somatization symptoms, but were not exclusively attributed to a psychiatric disorder.

Biological rhythms were assessed using the Biological Rhythms Interview of Assessment in Neuropsychiatry (BRIAN) scale. BRIAN consists of 18 items that measure sleep, social rhythm, general activities, and eating behavior. They range from one (difficulties in maintaining a usual biological rhythm) to four (severe challenges in maintaining a usual biological rhythm). Total scores ranged from 18 to 72, with higher scores indicating poorer biological rhythm (Giglio et al. 2009).

Psychosocial functioning was assessed using the Functional Assessment Short Test (FAST) scale. The FAST includes 24 items that assess six specific areas of functioning: autonomy, occupational functioning, cognitive functions, financial issues, interpersonal relationships, and leisure time. The options are determined by the sum of the items, which ranges from 0 (indicating no problem) to 3 (indicating severe limitations) (Cacilhas et al. 2009; Rosa et al. 2007). Trained and qualified health professionals conducted the interview.

### Physical characteristics and markers of oxidative stress

Height was measured in meters using a pocket stadiometer. Weight was measured in kilograms using a digital scale. Body mass index (BMI) was calculated using the formula kg/m^2.

The markers of oxidative stress investigated in this study included the following: non-protein thiol antioxidants (NPSH), protein thiols (PSH), total antioxidant capacity, advanced oxidation protein products (AOPP), MPO, thiobarbituric acid reactive substances (TBARS), ascorbic acid, and ROS (Borba et al. 2025). For blood sample collection, 6 mL of blood was drawn from each participant via venipuncture into an anticoagulant-containing tube. The blood was immediately centrifuged at 3,000 rpm for 10 min, and then 1.5 mL was aliquoted and frozen at −80 °C for subsequent biochemical analyses. In addition, serum was used immediately for the SARS-CoV-2 rapid antibody test (colloidal gold immunochromatography) following the manufacturer's instructions (Leccurate, Lepu Technology).

### Analysis of oxidative stress parameters

#### Determination of total thiol (PSH) and non-protein thiol (NPSH) levels

The protocol established by Ellman (1959) was used to determine both total thiol and non-protein thiol levels. This method involves reducing 5,5'-dithiobis (2-nitrobenzoic acid) (DTNB) and measuring the absorbance at 412 nm. For the total thiol assay, 40 µL of serum was added to a 96-well plate, followed by mixing with 200 µL of potassium phosphate buffer (PPB) (1 M, pH 6.8). Subsequently, 20 µL of DTNB was added, and the absorbance reading was performed immediately. In the case of non-protein thiols, the same experimental procedure was performed, except that the samples were deproteinized by adding an equal volume of 10% trichloroacetic acid (TCA) prior to analysis. Thirty microliters of supernatant were used for this analysis. The results were determined using a cysteine ​​standard curve and expressed in µM (Ellman et al. 1959).

#### Total antioxidant capacity

An antioxidant assay kit (Sigma.® Life Science, Darmstadt, Germany) was used to evaluate the total antioxidant capacity of the participants' serum. The principle of this antioxidant assay is based on the formation of a ferryl myoglobin radical from met-myoglobin and hydrogen peroxide, which oxidizes ABTS (2,2'-azino-bis (3-ethylbenzthiazoline-6-sulfonic acid)), resulting in the production of a radical cation (ABTS +), which is a soluble green chromogen. To perform the assay, 10 µL of the sample and 20 µL of the myoglobin solution were added to a 96-well plate, then mixed with 150 µL of the ABTS substrate solution. After 5 min of incubation at room temperature, 100 µL of the solution was added to the wells, and the absorbance was measured at 405 nm using a plate reader. A Trolox calibration curve (mM) was used as a reference standard antioxidant for comparison purposes (Borba et al. 2025

#### Advanced oxidation protein products (AOPP)

Advanced oxidation protein products (AOPPs) have been considered novel markers of protein oxidation and damage, primarily to albumin, that directly reflect protein function (Selmeci et al. 2005). Thus, 40 µL of serum sample was mixed with PBS (20 mM, pH 7.4) in a 96-well plate, and an initial reading was taken at 340 nm. Afterward, 20 µL of absolute acetic acid and 10 µL of potassium iodide (1.16 M in PBS) were added to the pre-reading wells, and a new reading was taken at the same wavelength. Finally, the results were calculated by subtracting the pre-reading absorbance from the final reading and expressed as µM chloramine T equivalents using a standard curve.

#### Myeloperoxidase (MPO) activity

In the assay, 12 µL of serum sample was mixed with 148 µL of AAP solution in phenol (AAP, 2.5 mM; phenol, 20 mM) and 17 µL of H_2_O_2_ solution (17 mM). This mixture was incubated at 37 ºC for 30 min, and the reading was subsequently performed spectrophotometrically. The results were expressed in μM of quinonimine per mg of protein produced in 30 min (μMq/mg/30 min). MPO is a heme enzyme produced in response to inflammatory mediators and released by leukocytes at the site of injury. Its presence reflects the activation of neutrophils and lymphocytes. MPO catalyzes the reaction between chlorine ions and H_2_O_2_, generating hypochlorous acid (HOCl), a reactive oxygen species that subsequently reacts to produce singlet oxygen and hydroxyl radical. To detect MPO activity, an oxidative coupling system of phenol and 4-aminoantipyrine (AAP) is used in the presence of H_2_O_2_ as an oxidizing agent. This reaction forms a colored product, quinonimine, whose maximum absorbance occurs at 492 nm (Suzuki et al. 1983).

#### Lipid peroxidation

Lipid peroxidation is a rapid reaction resulting from the breakdown of polyunsaturated fatty acids, usually measured by its products, mainly thiobarbituric acid reactive substances (TBARS), with malondialdehyde (MDA) as the main one (Jentzsch et al. 1996). To evaluate this product, the reaction of thiobarbituric acid (TBA) with serum samples was used; in the presence of MDA, it forms a pink product that can be read at 532 nm. Briefly, 20 µL of the samples were mixed with 55 µL of distilled water, 100 µL of orthophosphoric acid (0.2 M), and 25 µL of TBA (0.1 M). After 45 min of incubation at 37 °C, the spectrophotometric reading was taken. The results were expressed in nM MDA/mL.

#### Ascorbic acid content

The ascorbic acid content was determined using the method described by Jacques-Silva et al. (2001), with an absorbance reading at 520 nm. Briefly, 200 μL of serum samples were first deproteinized by adding an equal volume of 10% trichloroacetic acid (TCA). Afterward, 100 μL of the remaining supernatant was mixed with 25 μL of distilled water, 25 μL of TCA (13.3%), and 20 μL of 2,4-dinitrophenylhydrazine (DNPH), then incubated for 2 h at 37 °C. At the endpoint, a red–orange product was generated in proportion to the ascorbic acid content, which was expressed in µg/dL using the ascorbic acid standard (Jacques-Silva et al. 2001).

#### Detection of reactive oxygen species (ROS)

ROS formation was estimated by the fluorometric protocol established by Ali et al. (1992), in which 10 µL of serum was incubated with 100 µL of 2',7'-dichlorofluorescein diacetate (DCFH-DA, 7 μM). After 30 min of incubation at 37 °C, the final oxidation product of DCFH-DA, dichlorofluorescein (DCF), was measured. The fluorescence emission intensity was read at 525 nm emission and 488 nm excitation (Thermo Scientific™ Varioskan™ LUX). The results are expressed as a percentage of ROS (%) compared to the control (Ali et al. 1992).

### Statistical analysis

Statistical analyses were performed using SPSS 22.0. The normality of continuous variables was assessed using the Shapiro–Wilk test, and all variables were non-normally distributed. Thus, categorical variables were represented as absolute and relative frequencies and compared using the chi-square test. In contrast, continuous variables were presented as medians and interquartile ranges (IQRs), and group comparisons were assessed using the Mann–Whitney U test. Binary logistic regression was used to examine the association between total antioxidant capacity and current anxiety disorder (yes/no), adjusting for obesity, age, sex, race, years of schooling, marital status, occupation, and COVID-19, ABEP score. Results are presented as odds ratios (OR) and 95% confidence intervals (95% CI). To account for multiple comparisons among oxidative stress biomarkers, p-values were adjusted using the Benjamini–Hochberg false discovery rate (FDR) method. Statistical significance was set at p < 0.05. The results are presented in tables. Table 1 describes sociodemographic characteristics of the total sample. Table 2 compares clinical characteristics between individuals with anxiety disorders and individuals without anxiety disorders. Table 3 compares oxidative stress parameters between individuals with anxiety disorders and individuals without anxiety disorders.

**Table 1:** Sociodemographic characteristics of the sample

Characteristics	No anxiety disorders n = 234 n (%)/Median (IQR)	Anxiety disorders n = 116 n (%)/Median (IQR)	p-value
*Sex* Female Male	145 (61.9%) 89 (38.1%)	86 (74.1%) 30 (25.9%)	**0.024**
Age^1^	37.00 (27.00–51.00)	33.50 (25.00–33.50)	**0.035**
*Racial group* White Diverse Backgrounds Years of education^2^	196 (83.8%) 38 (16.2%) 16.00 (11.75–18.00)	92 (79.3%) 24 (20.7%) 15.00 (12.00–15.00)	0.305 0.981
*Relationship Status* Single In a Relationship	127 (54.3%) 107 (45.7%)	59 (50.9%) 57 (49.1%)	0.547
*EmploymentStatus* Engaged Non-engaged	224 (95.7%) 10 (4.3%)	102 (87.9%) 14 (12.1%)	**0.007**
Socioeconomic classification	18.04 (7.00–32.00)	17.13 (5.00–29.00)	0.079
*BMI Category*^*3*^ Obese Non-obese	3 (1.3%) 227 (98.7%)	5 (4.5%) 106 (95.5%)	0.067
*COVID-19* Yes No	30 (24.8%) 91 (75.2%)	13 (33.3%) 26 (66.7%)	0.295

^1^variable contains 1 missing data point in the no anxiety group

^2^variable contains 8 missing data points in the individuals without anxiety disorders and 2 in the individuals with anxiety disorders

^3^variable contains 9 missing data points in the anxiety group

*IQR* interquartile range, *BMI* body mass index

**Table 2:** Psychiatric symptoms among individuals with and without anxiety disorders

Psychiatric symptoms	No anxiety disorders n = 95 Median (IQR)	Anxiety disorders n = 36 Median (IQR)	p-value
HAM-D^1^	3.00 (1.00–5.00)	7.00 (4.00–11.00)	** < 0.001**
HAM-A^2^	4.00 (1.00–8.00)	11.00 (6.00–18.00)	** < 0.001**
Stress levels^3^ FAST^4^ BRIAN^5^	14.00 (7.00–24.00) 13.00 (7.00–21.00) 30.00 (26.00–38.00)	30.50 (19.00–40.00) 17.00 (11.00–25.00) 38.00 (32.50–43.00)	** < 0.001** **0.001** ** < 0.001**

^1^Variable contains 15 missing data in the individuals without anxiety disorders 5 missing in the individuals with anxiety disorders, ^2^variable contains 3 missing data in the individuals without anxiety disorders, ^3^variable contains 3 missing data in the individuals without anxiety disorders and 6 missing data in the individuals with anxiety disorders, ^4^variable contains 65 missing data in the individuals without anxiety disorders and 31 missing data in the individuals with anxiety disorders, ^5^variable contains 36 missing data in the individuals without anxiety disorders and 19 missing data in the individuals with anxiety disorders. Differences between groups were assessed using the Mann–Whitney U test

*HAM-D* Hamilton depression rating scale, *HAM-A* Hamilton anxiety rating scale, *FAST* functional assessment short test, *BRIAN* biological rhythms interview of assessment in neuropsychiatry, *IQR* interquartile range

**Table 3:** Biomarker levels among individuals with and without anxiety disorder

Baseline biomarkers	No anxiety disorders n = 95 Median (IQR)	Anxiety disorders n = 36 Median (IQR)	p-value	p (FDR)
NPSH^1^	0.71 (0.66–0.75)	0.71 (0.69–0.75)	0.112	0.146
PSH^2^	1.92 (1.61–2.47)	2.01 (1.51–2.37)	0.786	0.883
Total antioxidant capacity^3^ AOPP^4^ MPO^5^ ROS^6^ TBARS^7^ Ascorbic Acid^8^	0.64 (0.57–0.67) 3.01 (2.51–3.77) 1.52 (1.33–1.85) 2.86 (2.19–3.66) 25.11 (18.34–33.12) 14.74 (13.49–21.36)	0.60 (0.45–0.67) 3.07 (2.45–3.68) 1.61 (1.34–1.97) 2.91 (2.31–3.71) 24.82 (17.79–33.53) 14.56 (13.48–20.76)	**0.039** 0.788 0.364 0.889 0.975 0.687	0.312 0.883 0.631 0.889 0.975 0.883

^1^Variable contains 90 missing data in the no anxiety group and 41 in the anxiety group, ^2^variable contains 94 missing data in the individuals without anxiety disorders and 41 in the individuals with anxiety disorders, ^3^variable contains 119 missing data in the individuals without anxiety disorders and 53 missing data in individuals with anxiety disorders, ^4^variable contains 119 missing data in the individuals without anxiety disorders and 53 missing data in the individuals with anxiety disorders, ^5^variable contains 92 missing data in the individuals without anxiety disorders and 42 missing data in the individuals with anxiety disorders, ^6^variable contains 120 missing data in the individuals without anxiety disorders and 53 missing data in the individuals with anxiety disorders, ^7^variable contains 83 missing data in the individuals without anxiety disorders and 34 missing data in the individuals with anxiety disorders; ^8^variable contains 94 missing data in the individuals without anxiety disorders and 39 missing data in the individuals with anxiety disorders. Differences between groups were assessed using the Mann–Whitney U test

*IQR* interquartile range

## Results

The study included 350 individuals, with 234 individuals without anxiety disorders and 116 individuals with anxiety disorders. Sociodemographic characteristics are described in Table 1. No significant differences were observed between the groups regarding racial group (p = 0.305), years of education (p = 0.981), relationship status (p = 0.547), socioeconomic classification (p = 0.079), body mass index (BMI) (p = 0.295) or COVID-19 disease (p = 0.295). However, significant differences were observed in sex (p = 0.024), age (p = 0.035), and employment status (p = 0.007), with a higher proportion of females and of individuals who were professionally engaged in the groups. In addition, individuals with anxiety disorders were younger than individuals without anxiety disorders.

In the comparison of psychiatric symptom severity between groups (Table 2), individuals with anxiety disorders showed greater severity of depressive symptoms (p < 0.0001), greater severity of anxious symptoms (p < 0.0001), higher stress levels (p < 0.0001), greater dysfunction in biological rhythms (p < 0.001), and worse functionality (p = 0.001) compared to the individuals with no anxiety disorders.

Finally, the levels of predictive biomarkers are presented in Table 4. No significant differences were observed between the groups for NPSH, PSH, AOPP, MPO, ROS, TBARS, and ascorbic acid. Although total antioxidant capacity showed a nominal association (p = 0.039), this finding did not remain statistically significant after correction for multiple comparisons (p = 0.312). In addition, the adjusted model was significant (Omnibus test: χ^2^ = 22.988, p = 0.006) and showed adequate fit (Hosmer–Lemeshow test: χ^2^ = 8.596, p = 0.378; Nagelkerke R^2^ = 0.178). Higher total antioxidant capacity values were associated with lower odds of current anxiety disorder (OR = 0.058, 95% CI 0.006–0.599, p = 0.017). Age was inversely associated with current anxiety disorder (OR = 0.951, 95% CI 0.918–0.985, p = 0.005). Sex was also associated with current anxiety disorder (OR = 0.399, 95% CI 0.187–0.851, p = 0.017). No significant associations were found for obesity, race, years of schooling, marital status, occupation, COVID-19, or ABEP score.

## Discussion

### Sociodemographic characteristics

According to the results, most sociodemographic characteristics showed no statistically significant differences between the individuals with and without anxiety disorders (race, years of education, relationship status, socioeconomic classification, COVID-19 disease, and body mass index). However, no significant differences were observed between individuals with and without anxiety disorders regarding COVID-19 infection. This finding suggests that, within our sample, prior exposure to SARS-CoV-2 may not have had a direct impact on anxiety outcomes. Although previous studies have reported increased psychological distress associated with the pandemic context, our results indicate that COVID-19 infection per se was not a determining factor. It is important to consider that the broader psychosocial consequences of the pandemic, rather than infection status alone, may play a more relevant role in mental health outcomes. The findings suggest that anxiety disorders are more frequent among women, younger individuals, and those who are professionally engaged. This pattern may reflect known demographic and social vulnerabilities associated with anxiety, as reported in previous studies.

Regarding sex-based differences, the literature consistently demonstrates a higher predisposition to anxiety and stress-related disorders among women compared to men (Bernal Arenas et al. 2025; Nolting et al. 2025). Terlizzi and Zablotsky (2024) further emphasized that, whether at mild, moderate, or severe levels, women are more affected than men. Moreover, Women appear to exhibit greater difficulties in emotional regulation in response to stress (Chaudhary et al. 2024; Macías-Espinoza et al. 2022). Studies consider the influence of brain structures, as well as hormonal and genetic factors, that increase the risk of anxiety disorders in women (Farhane-Medina et al. 2022; Kundakovic and Rocks 2022).

Interestingly, our findings showed that employed individuals exhibited higher rates of psychological distress than those who were unemployed. In general, employment is considered a protective factor for mental health (Aanesen et al. 2024; Sterud et al. 2025; Yang et al. 2024). Nevertheless, rates of stress and occupational burnout have been increasing substantially (Amellah et al. 2025b, a; García-Pérez et al. 2025; Lee et al. 2025). It is possible that social demands for performance, excessive targets, task overload, and negative work environments contribute to this scenario (Lukan et al. 2022; Tjugum et al. 2025). The growing reality of burnout has drawn increasing attention in research and within public health (Tang et al. 2025). Furthermore, the overlap between professional responsibilities and other social roles may compromise a healthy work–life balance, thereby contributing to the accumulation of occupational stress (Yuan et al. 2023).

Consistent with our findings, previous studies have reported a correlation between higher resilience levels and greater life experience (Feliciano et al. 2022; Höltge et al. 2021). Additionally, Mikneviciute et al. (2023) reported that older individuals exhibited lower total cortisol levels. The same was observed for stress-induced cortisol (Mikneviciute et al. 2023). Thus, both resilience levels and stress management appear to be positively influenced by age. This may partly explain the lower anxiety levels observed among older individuals.

### Psychiatric symptoms

Our study revealed that individuals with anxiety exhibited higher levels of depressive, anxiety, and stress symptoms compared to those without anxiety (Table 2*).* This can be explained by several factors, including dysregulation of the hypothalamic–pituitary–adrenal axis (HPA) (Juruena et al., 2020; Rana et al. 2022; Tong et al. 2023). When confronted with danger, the HPA axis is activated. Subsequently, the hypothalamus secretes corticotropin-releasing hormone (CRH), which in turn stimulates the pituitary gland to release adrenocorticotropic hormone (ACTH). Ultimately, the release of cortisol into the bloodstream marks the conclusion of the physiological stress response (Ferraú et al. 2021; Leistner & Menke, 2020). Furthermore, depression and anxiety share substantially overlapping physiological mechanisms (Fabrazzo et al. 2021). Individuals with anxiety disorders tend to display heightened sensitivity to environmental and psychological stressors, which may impair emotion regulation processes and contribute to exaggerated stress reactivity (Mac et al. 2024; Khalkhali et al. 2024). Thus, it is understandable that individuals without anxiety disorders exhibit healthier responses to stress and anxiety, as well as lower rates of depressive symptoms, when compared to those with anxiety disorders.

It is well established that higher levels of anxiety symptoms are associated with a greater tendency toward negative thoughts and cognitive rumination (Edgar et al. 2024). Ruminative thinking is likewise central to the maintenance of depression (Kovács et al. 2020). Similarly, excessive worry is present across levels of stress, and the greater the distress, the higher the risk to overall mental health (Gori and Topino 2021). Many individuals with anxiety will eventually develop depression, just as individuals with depression either suffer from or will develop anxiety disorders (Demyttenaere, Heirman 2020). The reasons for this coexistence are not yet fully understood; however, it is possible that genetic factors and similar neural and cognitive mechanisms are involved (Zlomuzica et al. 2025).

### Psychosocial functioning and biological rhythm

When analyzing biological rhythms in this study, alterations were more pronounced in the individuals with anxiety disorders. The data are consistent with our hypotheses.

It is well established that the circadian clock regulates the physiological perception of “day and night,” ensuring greater energy production during daytime and promoting organic repair during nighttime rest (Dollish et al. 2024). In this regulation, glucocorticoids play an essential role, influencing various physiological functions, disseminating the biological rhythm throughout the body (Androulakis 2021). Data show that glucocorticoid levels peak shortly after waking, a phenomenon observed in both diurnal and nocturnal animals (McEwen, Karatsoreos 2022). Such disruption can impair immune function, metabolism, and contribute to the development of physiological diseases and psychological disorders (Dollish et al. 2024). Likewise, elevated stress levels can alter circadian rhythms and cause reversible or permanent damage (Agorastos, Olff 2021). Alternatively, persistent changes in sleep patterns are associated with increased cortisol levels (McEwen, Karatsoreos 2022), activation of the sympathetic nervous system, inflammatory reactions, and stress responses (Yang et al. 2022).

In clinical manifestations of pathogenic anxiety, insomnia is a common symptom (American Psychiatric Association 2022). Indeed, hyperarousal, frequent worries, and ruminative thoughts are frequently associated with sleep loss (Edgar et al. 2024; Riemann et al. 2025). In agreement, Harvey (2005) reports that patients with insomnia exhibit sleep disturbances that lead to hyperarousal, psychological distress, and anxiety. Another common symptom in anxiety disorders is appetite alteration (American Psychiatric Association 2022). The hypothalamus is the primary regulator of hunger and satiety (Kuckuck et. al. 2022). Glucocorticoids, in turn, act on the hypothalamus, both directly and indirectly, to maintain this homeostasis (Meye, Adan 2014). However, stress can cause appetite disturbances, leading to either a loss or an increase in appetite. Acute stress, for instance, may result in appetite suppression. Personal and environmental factors, as well as the duration of stress, influence the course of this symptomatology (Sominsky, Spencer 2014).

As expected, psychosocial functioning was statistically significant. Indeed, the diagnosis of anxiety disorders is associated with occupational, social, and functional impairments, as well as with disruptions in other important areas of life (Song et al. 2025; Szuhany, Simon 2022). Moreover, anxiety symptoms per se are linked over time to impairments in functional and social performance, as well as overall quality of life, with greater symptom severity leading to more pronounced impairments (Dickson et al. 2024; Monira Alwhaibi 2025; Wilmer et al. 2021). Likewise, genetic variants related to the severity of anxiety symptoms substantially overlap with those associated with functional impairments (Skelton et al. 2025). Genetic factors that predispose to avoidance behaviors inherent to anxiety also strongly influence daily functioning (Ohi et al. 2025). thereby partially explaining the correlation between them.

### Oxidative stress

Among the oxidative stress biomarkers evaluated, only total antioxidant capacity demonstrated statistical significance. Participants in the anxiety group exhibited significantly reduced levels of this marker compared to those in the non-anxiety group (Table 3). However, the lack of significance after correction for multiple testing suggests that the observed differences in oxidative stress markers should be interpreted with caution and considered exploratory.

One of the main factors responsible for maintaining the body's vital dynamic is known as 'redox equilibrium´. This system regulates the simultaneous production and elimination of reactive species within the organism, helping to prevent the excessive buildup of harmful substances (Cecerska-Heryć et al. 2022; Sies 2021). In a healthy system, oxidative reactions are balanced by antioxidant agents. However, when antioxidant defenses are insufficient to manage this imbalance, the resulting toxicity can lead to various physical and psychological effects (Cecerska-Heryć et al. 2022). The brain is particularly susceptible to such damage because of its high oxygen demand and significant production of reactive species. For this reason, many studies have examined the connection between oxidative stress and psychiatric conditions, including anxiety disorders (Salim 2014; Negah and Forouzanfar 2024).

Neuroinflammation is also known to play a crucial role in oxidative damage. Cells of the central nervous system, such as astrocytes and microglia, can initiate this inflammatory response (Teleanu et al. 2022). Chronic stress exposure and psychiatric disorders in general are likewise associated with elevated levels of inflammation. Similarly, the harmful effects of recurrent stress on cells impact both the central and peripheral nervous systems (Bauer, Teixeira 2018; Westfall et al. 2024). Chronic stress exposure also compromises the immune system. As these impairments persist over time, the risk of developing psychiatric disorders, particularly depression and anxiety, increases (Westfall et al. 2024).

Regarding oxidative stress markers, Bolos et al. (2024) analyzed salivary cortisol levels in a group of students to investigate their relationship with stress and total antioxidant capacity. The authors demonstrated that although cortisol variations were not statistically significant, total antioxidant capacity decreased substantially during periods of heightened stress (Bolos et al. 2024). Several studies have also linked higher levels of total antioxidant capacity to a lower prevalence of depression (Liu et al. 2024, 2023; Zhao et al. 2022). Given these findings and the well-established link between oxidative stress, inflammation, and anxiety disorders, it is unsurprising that individuals without anxiety disorders exhibit lower total antioxidant capacity. However, a review of the scientific literature revealed no other studies specifically linking the markers examined in this study to anxiety disorders.

The data presented highlight the complexity of factors associated with anxiety. Research that investigates this disorder in depth, examining its predictors and comorbidities, is of great importance. Our study had limitations, including the subjective nature of symptom assessment, the heterogeneity of anxiety disorders, the limited generalizability of the findings (as the sample may not fully represent broader or more diverse populations), and the complexity of analyzing oxidative stress markers. In addition, the lack of detailed information on participants’ exposure to pharmacological and/or psychotherapeutic interventions during the study period is an important limitation. Such treatments may substantially influence both symptom trajectories and biological markers, potentially confounding the observed associations. Therefore, the findings should be interpreted with caution. Future investigations using larger, more clinically homogeneous, and more demographically diverse samples, along with systematic assessment and control of treatment-related variables, a comprehensive panel of biomarkers, and direct interventions targeting anxiety predictors, may prove particularly valuable.

In conclusion, our findings support an exploratory role of oxidative stress in the pathophysiology of anxiety disorders, especially in contexts of heightened vulnerability such as the COVID-19 pandemic. Therefore, the results highlight the importance of integrated approaches that address both psychological and biological factors in the management of anxiety disorders, as well as the need for further research exploring interventions targeting oxidative stress.

## Data Availability

No datasets were generated or analysed during the current study.
